# A Nomogram for the Prediction of Prognosis in Patients With Distant Metastases of Nasopharyngeal Carcinoma

**DOI:** 10.3389/fonc.2019.00240

**Published:** 2019-04-05

**Authors:** Liang Zhao, Qiuming Lin, Jianwei Gu, Huan Zhang, Haojun Chen, Qin Lin

**Affiliations:** ^1^Department of Radiation Oncology, Xiamen Cancer Hospital, The First Affiliated Hospital of Xiamen University, Teaching Hospital of Fujian Medical University, Xiamen, China; ^2^Department of Nuclear Medicine and Minnan PET Center, Xiamen Cancer Hospital, The First Affiliated Hospital of Xiamen University, Teaching Hospital of Fujian Medical University, Xiamen, China

**Keywords:** nasopharyngeal carcinoma, distant metastases, prognosis, nomogram, overall survival

## Abstract

**Background:** Patients with metastatic nasopharyngeal carcinoma (NPC) have heterogeneous survival outcomes. This study aimed to establish an effective prognostic nomogram for patients with NPC with distant metastases using easily determined factors.

**Methods:** The nomogram was based on a retrospective study of 103 patients with metastatic NPC at the First Affiliated Hospital of Xiamen University during January 2009–March 2016. Nomogram performance was evaluated using a concordance index (C-index) and assessed using calibration plot. Bootstraps with 1,000 resamples were applied to these analyses.

**Results:** In univariate and multivariate Cox proportional hazards model analyses, chemotherapy, metastatic liver involvement, number of tumor metastases, N stage and derived neutrophil–lymphocyte ratio correlated with overall survival (OS). The recurrence probability calibration curve indicated good agreement between nomogram-based predictions and actual observations. For OS predictions, the nomogram had a C-index of 0.824 (95% confidence interval, 0.74–0.91). The stratification by nomogram score of patients into different subgroups showed significant distinction.

**Conclusion:** This novel nomogram comprises factors that are easily determined at most hospitals and can predict survival in patients with distant metastases of NPC. This model can precisely estimate the survival of individual patients and identify subgroups of patients requiring specific therapeutic strategies.

## Introduction

Nasopharyngeal carcinoma (NPC), which is endemic in Southern China and Southeast Asia, has a unique geographical distribution pattern (1). Advances in radiotherapy and the broad application of chemotherapy in recent decades have yielded great improvements in the 5-year overall survival (OS) of affected patients. However, distant metastasis of NPC remains a key treatment obstacle. Specifically, 17–54% of patients with NPC experience treatment failures due to distant metastases, and these patients have disappointing outcomes (2–4).

The role of chemotherapy for metastases of NPC, a highly chemosensitive malignancy, has been well established. Zhang et al. recently demonstrated that chemotherapy with gemcitabine plus cisplatin could significantly improve progression-free survival (PFS) among patients with metastatic NPC (5), thus establishing this regimen as a standard first-line treatment option for these patients (5). Although the systemic treatment options of patients with metastatic NPC have gradually evolved to include other chemotherapeutic regimens, targeted therapy, and immunotherapy, the outcomes remain heterogeneous.

The American Joint Committee Cancer (AJCC) tumor–node–metastasis (TNM) staging system is currently the most widely used staging strategy and is a fundamental determinant of prognostic predictions. However, the usefulness of this system for patients with metastatic NPC is limited, as the clinical outcomes differ even among patients with the same stage who receive similar treatment regimens (6). Many additional factors affecting the prognosis of NPC have since been identified, including the Epstein–Barr virus (EBV) DNA concentration, miRNAs and the derived neutrophil-lymphocyte ratio (dNLR) (7–9). A scoring system that incorporates several of these factors would likely help to direct individualized patient treatments.

Nomograms are considered reliable for risk quantification. These tools quantify risk by incorporating and illustrating important factors related to oncologic prognosis. Nomograms have been proven to generate more precise predictions for several types of cancers when compared to the conventional TNM staging systems (10, 11). However, few nomograms are available for predicting the long-term survival outcomes of patients with NPC with distant metastases. In this study, we aimed to combine the TNM staging system, metastatic sites, number of metastases, dNLR and other independent factors into a nomogram for NPC patients with distant metastases. Such a nomogram could potentially enable clinicians to precisely calculate the survival outcomes of individual patients with distant metastases of NPC.

## Patients and Methods

### Patients

Between January 2009 and March 2016, 791 patients with newly pathologically diagnosed and previously untreated NPC were retrospectively reviewed. Among them, 120 patients initially presented with or developed metastatic NPC before March 2016. The following enrolment criteria were applied to subjects of this retrospective study: (i) complete sociodemographic data and laboratory test results; (ii) complete imaging data [magnetic resonance (MR)/computed tomography (CT) of the nasopharynx and neck, technetium-99m (^99^Tc^m^-MDP) bone scans, MR/CT/ultrasound of the liver, chest CT and/or whole-body ^18^F-fluorodeoxyglucose (FDG) positron emission tomography (PET)/CT]; (iii) pathologically confirmed World Health Organization (WHO) type II or WHO type III NPC; (iv) pathologically or radiologically confirmed distant metastatic lesion(s) and (v) a Karnofsky performance status (KPS) score ≥70. The following exclusion criteria were also applied: (i) brain metastases; (ii) other types of malignancy; and (iii) serious renal or liver disease requiring treatment.

Data were retrieved for 120 patients. Of those, 17 patients were excluded from the total score analysis because of missing laboratory data or a lost to follow-up status. Finally, 103 patients were deemed eligible for risk stratification. The tumors were staged according to the 2009 AJCC staging system. This study was approved by the Clinical Research Ethics Committee of the First Affiliated Hospital of Xiamen University.

### Treatment and Follow-Up

All patients received multimodal treatment after diagnosis. The first-line regimen comprised platinum-based chemotherapy for 4–6 cycles according to our institutional experience. Patients who could not tolerate or were unwilling to receive additional chemotherapy were administered other therapies, such as palliative radiotherapy, targeted therapy, and surgery. For each patient, treatment was defined according to the experience of our hospital and the wishes of the individual patient.

Follow-up examinations were performed every 3 months during the first and second years after treatment and every 6 months thereafter according to the standard practice of our hospital. These examinations included nasopharyngeal and neck MR imaging, nasopharyngoscopy, chest CT, MR/CT/ultrasonography of the liver, complete blood cell counts, blood biochemical testing, and a ^99^Tc^m^-MPD bone scan. OS was defined as the duration from the date of the most recent metastasis diagnosis to the date of death from any cause or censorship on the last follow-up date (March 31, 2016).

### Construction of the Nomogram

Univariate and multivariate analyses were performed using the Cox proportional hazards model. The age at metastasis; sex; chemotherapy (< 2 vs. ≥2 cycles); radiotherapy; targeted therapy; metastases of the liver, lung and/or bone metastasis; number of tumor metastases; synchronous or metachronous status; body mass index (BMI); smoking history; pretreatment dNLR (low vs. high) at the time of metastasis; and stages of the primary tumor (T1 or T2 vs. T3 or T4) and regional lymph nodes (N1–N2 vs. N3) at the initial diagnosis were included in the univariate regression models. Factors identified as significant predictors of OS in the univariate analysis were subsequently entered into the multivariable analyses via the Cox regression model. The cut-off values for continuous variables were determined based on the receiver operating characteristic (ROC) curves. The χ2, χ2 continuity correction and Fisher's exact test were used to determine the proportion of independents. Statistical analyses to identify independent prognostic factors were conducted in SPSS 22.0 (IBM, Armonk, NY, USA). On the basis of the results of the multivariable analysis, a nomogram was formulated by R version 3·1.1 statistical analysis software (http://www.r-project.org).

### Validation and Calibration of the Nomogram

Following the above analyses, a nomogram was developed based on the multivariate Cox regression results. The final prediction model used for the nomogram was selected using a backward stepdown procedure with a threshold *P* of < 0.05. The nomogram performance was evaluated using a concordance index (C-index) and assessed using a calibration plot; bootstraps with 1,000 resamples were applied to both analyses. The total points for each patient in the validation cohort were calculated using the established nomogram, after which a Cox regression analysis of the whole cohort was performed using the total points as a factor. The C-index and calibration curves were derived based on the regression analysis.

### Risk Stratification Based on the Nomogram Beyond TNM staging

In order to demonstrate the independent discrimination ability of the prognostic nomogram beyond standard TNM staging, we determined the cut-off values by grouping all patients evenly into different risk groups according to the total risk scores in the study cohort. Survival curves for different risk groups were generated using the Kaplan-Meier estimates and were compared using the log-rank test.

## Results

### Patient Characteristics

The 103 patients included in this analysis comprised 83 men and 20 women with a median age at metastasis of 50 years (range, 23–82 years). All patients had histologically confirmed non-keratinizing undifferentiated or low-keratinizing squamous cell cancer (WHO II or WHO III). Additionally, 66% (68) of patients had stage N1–2 disease, while 34% (35) had stage N3 disease. Forty-three patients (41.7%) presented with liver metastases. The median number of tumor distant metastases was 10 (range, 1–26), and the median pretreatment dNLR at metastasis was 2.33 (range, 0.67–8.52; Table 1). Eighty-seven patients (84.5 %) died after a median follow-up of 16 months (range, 1–79 months).

**Table 1:** Clinical characteristics of the study patients.

**Variable**	**Number**	**%**
**AGE (YEARS)**		
Median	50 (23–82)	
<50	48	46.60
≥50	55	53.40
**SEX**		
Male	83	80.60
Female	20	19.40
**T STAGE**		
T1/T2	19	18.45
T3/T4	84	81.55
***N*** **STAGE**		
N1–N2	68	66
N3	35	34
**TREATMENT**		
Chemotherapy (≥2 cycles)	64	62.10
Radiotherapy	48	46.60
Target therapy	18	17.50
**SITE OF METASTASIS**		
Liver metastasis	43	41.70
Lung metastasis	41	39.80
Bone metastasis	75	72.80
**NUMBER OF METASTASES**		
Median	10 (1–26)	
Synchronous	33	32.04
Metachronous	70	67.96
**HISTOLOGY, WHO TYPE**		
II	30	29.10
III	73	70.90
**SMOKING HISTORY**		
Non-smoker	64	62.10
Smoker	39	37.90
**BMI (kg/m**^**2**^**)**		
<18.5	27	26.20
18.5–23.9	65	63.10
≥23.9	11	10.70
**dNLR**		
Median	2.33 (0.67–8.52)	

*T, tumor; N, node; BMI, body mass index; dNLR, derived neutrophil–lymphocyte ratio*.

### Independent Prognostic Factors

Initially, the covariates listed in Table 1 were analyzed using a Cox univariate factor regression model, which identified chemotherapy cycles (*P* < 0.001), liver metastasis (*P* < 0.001), number of tumor metastases (*P* < 0.001), N stage(*P* = 0.045), and dNLR (*P* < 0.001) as factors significantly associated with OS. By contrast, the age at metastasis (*P* = 0.532), sex (*P* = 0.178), T stage (*P* = 0.074), radiotherapy (*P* = 0.202), targeted therapy (*P* = 0.102), lung metastasis (0.774), bone metastasis (*P* = 0.164), synchronous metastasis (*P* = 0.065), histology (*P* = 0.176), smoking history (*P* = 0.878), and BMI (*P* = 0.579) were not found to correlate with OS (Table 2). All significant factors from the univariate analysis were entered into the Cox regression-based multivariate analysis (Table 3). Chemotherapy cycles (*P* < 0.001), liver metastasis (*P* < 0.001), number of tumor metastases (*P* < 0.001), N stage(*P* = 0.001), and dNLR (*P* = 0.011) remained independent prognostic factors in the Cox model.

**Table 2:** Univariate analysis of the Cox risk ratio model for OS.

**Variable**	**Hazard ratio**	**95% CI**	***P-*value**
Age (years)	0.86	0.54–1.38	0.532
<45			
≥45			
Sex	1.43	0.85–2.42	0.178
Male			
Female			
T stage	1.86	1.09–3.18	0.074
T1–2			
T3–4			
N stage	0.64	0.41–0.99	**0.045**
N1–2			
N3			
Chemotherapy	2.21	1.44–3.40	** <0.001**
<2 cycles			
≥2 cycles			
Radiotherapy	1.32	0.86–2.02	0.202
Yes			
No			
Target therapy	1.67	0.90–3.07	0.102
Yes			
No			
Liver metastasis	0.39	0.25–0.61	** <0.001**
Yes			
No			
Lung metastasis	1.07	0.69–1.66	0.774
Yes			
No			
Bone metastasis	0.70	0.43–1.15	0.164
Yes			
No			
Number of metastases	0.26	0.16–0.42	** <0.001**
<8			
≥8			
dNLR	0.46	0.30–0.71	** <0.001**
<2.6			
≥2.6			
Synchronous	0.64	0.40–1.01	0.055
Yes			
No			
Histology, WHO type	1.37	0.87–2.16	0.176
II			
III			
Smoking history	1.04	0.67–1.60	0.878
Yes			
No			
BMI (kg/m^2^)	0.87	0.53–1.43	0.579
<18.5			
≥18.5			

*OS, overall survival; T, tumor; N, node; BMI, body mass index; dNLR, derived neutrophil-lymphocyte ratio; CI, confidence interval. The bold values indicates p < 0.05*.

**Table 3:** Multivariate analysis of the Cox risk ratio model for OS.

**Variable**	**HR**	**95% CI**	***P*-value**
Chemotherapy	2.72	1.73–4.26	<0.001
<2 cycles			
≥2 cycles			
Number of metastases	0.23	0.13–0.40	<0.001
<8			
≥8			
Liver metastasis	0.34	0.21–0.54	<0.001
Yes			
No			
N stage	0.45	0.28–0.71	0.001
N1–2			
N3			
dNLR	0.56	0.36–0.88	0.011
<2.6			
≥2.6			

*OS, overall survival; HR, hazard ratio; CI, confidence interval; T, tumor; N, node; dNLR, derived neutrophil-lymphocyte ratio*.

### Nomogram for Predicting OS in Patients With Distant Metastases of NPC

**Figure 1:**
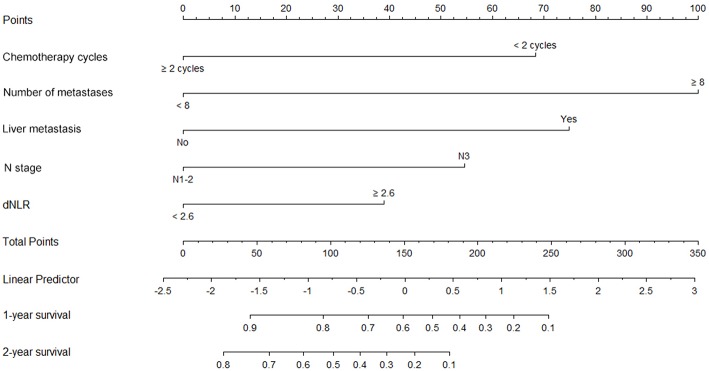
Nomogram A for predictions of 1- and 2-year overall survival (OS) in patients with metastatic nasopharyngeal carcinoma. This nomogram, which includes chemotherapy, the number of metastases, liver metastasis, N stage and dNLR, allows the user to determine the probability of the 1-year and 2-year OS for an individual patient using a combination of covariates. Using the patient's N stage, a line can be drawn straight upward to the “Points” axis to determine the associated score. After repeating the process for each variable, the scores for each variable can be summed and plotted on the “Total Points” axis. Finally, a vertical line can be drawn straight down from the plotted total point axis to the survival axis to determine the 1- and 2-year OS probabilities.

A nomogram incorporating the significant prognostic factors was established (Figure 1). Here, the number of distant metastases and presence of liver metastases made the largest prognostic contribution, followed by the number of chemotherapy cycles and N stage. By contrast, the dNLR level had a moderate impact on survival. Within these variables, each subtype was assigned a score on the point scale. Accordingly, by locating the summed total score on the total point scale, we could easily draw a straight line to determine the estimated probability of survival at each time point.

### Calibration and Validation of the Nomogram

**Figure 2:**
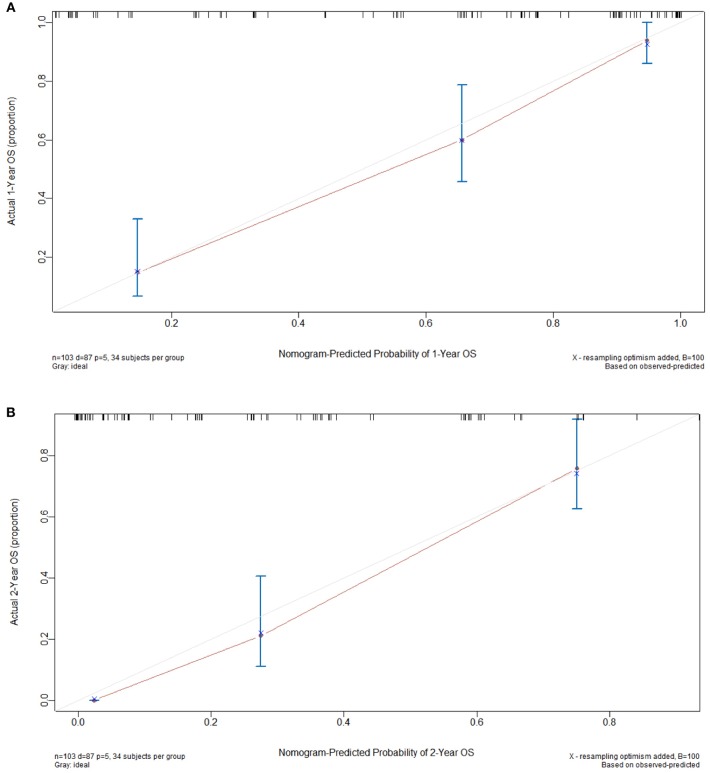
Calibration curves used to compare the nomogram-predicted and actual survival probabilities at 1 **(A)** and 2 years **(B)**. The actual overall survival (OS) is plotted on the y axis, while the nomogram-predicted probability is plotted on the x axis. The dotted line indicates the reference (i.e., ideal prediction).

The constructed nomogram included all independent prognosticators of 1- and 2-year OS identified in the multivariable analysis (Figure 1). The C-index for predicting OS was 0.824 [95% confidence interval (CI), 0.74–0.91]. A calibration plot of the survival probabilities at 1 (Figure 2A) and 2 years (Figure 2B) revealed good agreement between the nomogram-based prediction and the actual observation.

### Prognostic Nomogram for Risk Stratification

**Figure 3:**
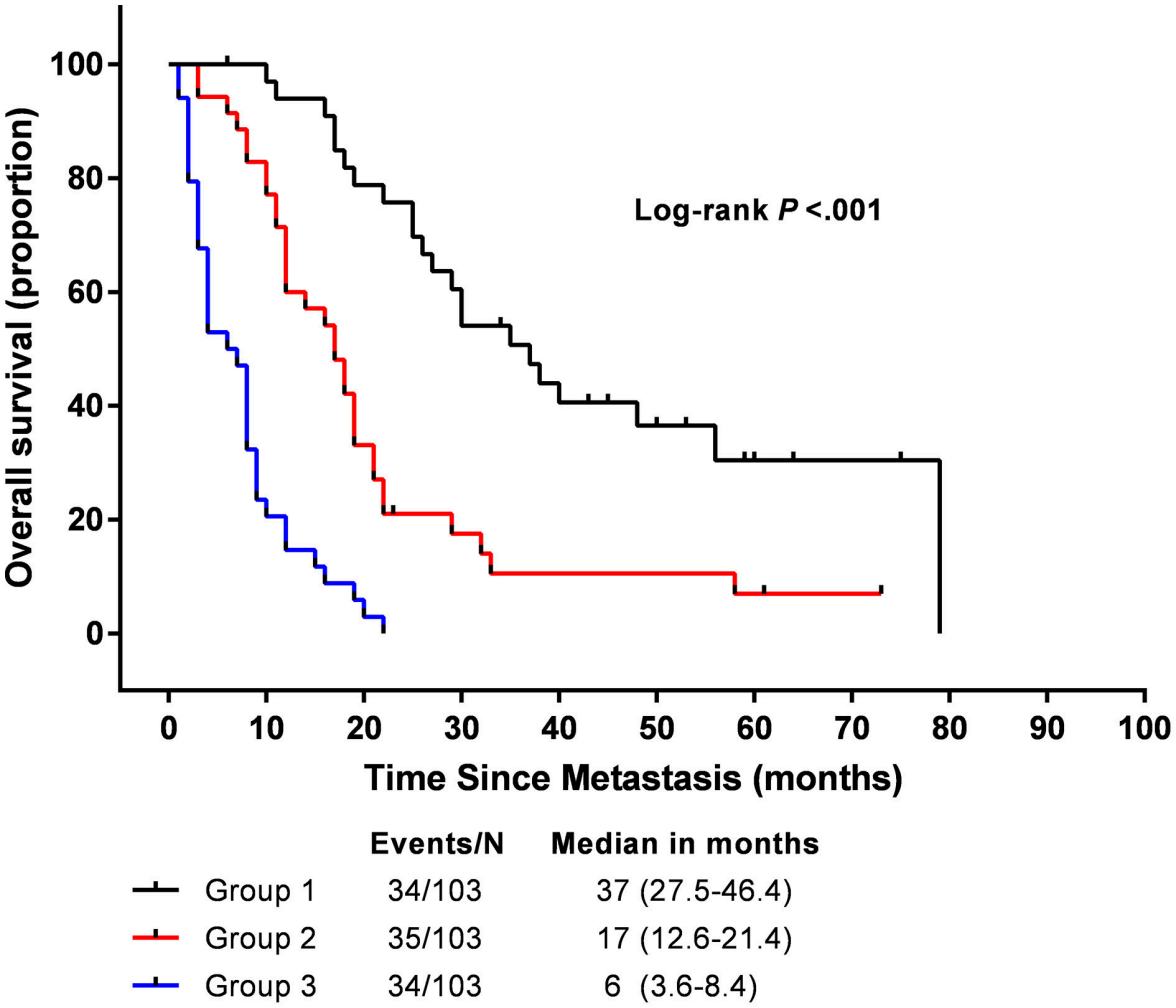
Overall survival in the subgroup according to a tertiles of the total score from nomogram.

We determined the cut-off values by grouping all patients in the study cohort into three subgroups based on the tertiles of total scores, each group represents a distinct prognosis. The Kaplan-Meier survival curves were subsequently delineated and were shown in Figure 3. Group 1 (total points 0–108, 34 patients) had the highest overall survival as 90.9 % for 1 year and 69.7 % for 2 years, respectively; followed by Group 2 (total points 108–199, 35 patients) as 60.0 and 17.5% for 1 and 2 years, respectively; Group 3(total points 199–338, 34 patients) showed the lowest overall survival as 14.7 and 0% for 1 and 2 years, respectively. The median OS in Group 1–3 are 37 (95%CI, 27.5–46.4), 17 (95%CI, 12.6–21.4), and 6 (95%CI, 3.6–8.4) months, respectively. Significant distinction for survival outcomes was observed between the three groups.

## Discussion

Among all head and neck cancers, NPC exhibits the highest propensity for distant metastasis (2). However, far fewer studies have evaluated patients with distant metastases of NPC, compared to their counterparts with non-metastatic advanced NPC. The metastatic NPC patients typically have an OS duration of < 15 months (12, 13). Accordingly, many patients diagnosed with metastatic NPC and their clinicians often have negative attitudes regarding treatment. Interestingly, it has been reported that a subset of patients with distant metastases of NPC still experience good OS outcomes in response to an aggressive therapy regimen (14, 15). However, we lack a reliable method of predicting which individuals are likely to get benefit from a more intensive treatment while avoid overtreatment in the unfavorable subgroup. The eighth edition of the AJCC TNM classification represents the most widely used staging system, in which patients with NPC are stratified according to tumor size and invasion, lymph node involvement, as well as distant metastasis. However, survival of patients with metastatic NPC varies widely. This may partly due to the current M staging system is purely based on whether the patient has distant metastasis, and all M1 patients are classified as clinical IVB stage according to the current AJCC staging system. Furthermore, the M classification from the previous AJCC staging system has never been modified for subdividing. As a result, this traditional staging system does not completely reflect the biological heterogeneity of metastatic NPC patients, and other independent risk factors are not taken into account in current AJCC staging systems. Therefore, a reliable prognostic method is needed, as this would enable the administration of individualized therapies to distinct subgroups of patients.

Several studies have attempted to build prognostic models for patients with metastatic NPC. For example, Ong et al. designed a prognostic index score (PIS) system based on liver and lung metastasis, anemia, a poor performance status, distant metastasis at initial diagnosis and the disease-free interval (16). However, that study assessed patients between January 1994 and December 1999 (16), and the availability of chemotherapeutic drugs and approaches to radiotherapy have since been modified during the era of intensity-modulated radiation therapy. In 2012, Jin et al. constructed a prognostic score model (PSM) that incorporated circulating tumor markers of metastatic NPC, performance status, age, hemoglobin level, lactate dehydrogenase (LDH) level, alkaline phosphatase (ALP) level and EBV DNA level (17). However, that scoring system may not be sufficiently precise, as each included factor received a score of 1 or 3 according to the n value (17).

In contrast to other systems, a nomogram can provide a visual representation of the results of a Cox model and facilitate individualized predictions for many cancers (10, 11, 18). However, a nomogram had not previously been developed to include both synchronous and metachronous metastatic NPC. As far as we know, this is the first study to develop a survival prognostic nomogram for this population. Using data from our study cohort, we built a nomogram predictive of OS among patients with metastatic NPC that was based on independent prognostic factors, including the numbers of chemotherapy cycles and metastases, occurrence of liver metastasis, N category and dNLR. Each of these factors is easily obtained at most hospitals. In addition, by stratifying patients into three risk groups from nomogram total score, we separated patients with distinct survival outcomes. We further note that our study cohort comprised patients at the First Affiliated Hospital of Xiamen University in Southern China, a region considered endemic for NPC. The unique geographic distribution of the patients and the reasonable sample size guarantee that our results are generally representative of Chinese patients with NPC.

We identified the independent prognostic factors for OS that were included in our nomogram through a univariate analysis and subsequent multivariate analysis. In the nomogram we established, the presence of liver metastases and the number of distant metastases made the largest prognostic contribution. Distant metastases of NPC most frequently involved the bone, lung and liver. Some studies have reported an association of hepatic invasion with poor survival in patients with NPC (12, 16, 19). In our study, we identified hepatic involvement as an important predictor of survival, which is consistent with previous reports. Regarding the number of metastatic lesions, most clinicians are only concerned with the distinction between oligometastasis and non-oligometastasis, as the former has been associated with a more favorable OS compared to widespread metastases (17, 20). However, it should be noted that some patients with multiple metastatic lesions still achieved relatively good survival outcomes. Therefore, it is important to clarify the relationship between the number of metastases and long-term survival. In our study, we used a ROC analysis to calculate the most discriminative cut-off value of the number of metastatic lesions and found an association of fewer than 8 metastases with better clinical outcomes. A similar study by Tian (21) used a cut-off number of 6 metastatic lesions to further confirm the relationship between a higher number of metastases and poorer survival.

Chemotherapy has also been selected as a candidate factor in this study because it has been recommended as a routine treatment for advanced NPC worldwide. However, the efficacy of chemotherapy regimens remains controversial, and a considerable number of publications have addressed this issue (22–24). In our study, the patients who received more than 2 cycles of chemotherapy were associated with a better OS. Another prognostic factor we selected from metastatic NPC is the N stage, this is because most NPC patients present with neck lymph node metastasis at the time of the initial diagnosis, and emerging evidence suggests that lymph node metastasis increases the risk of metastatic seeding of distant organs and correlates with an unfavorable prognosis (25, 26). Consistent with previous reports, our study further supports the relationship between the higher N stage and the poorer clinical outcome.

In-depth studies of the tumor–inflammation link have identified several blood markers as potential indicators of systemic inflammation and predictors of prognosis in patients with cancer, including the dNLR, C-reactive protein, albumin and LDH. The pretreatment dNLR, which reflects both the neutrophil and lymphocyte counts, can be easily determined in daily clinical practice via peripheral blood testing. A previous study of more than 12,000 patients has also supported the relationship between the higher value of dNLR and poorer OS outcome in different types of cancers (27). One of the possible reasons is that neutrophils can inhibit activated T cells and NK cells to induce immune suppression, while lymphocytes can inhibit tumor cell proliferation and metastasis via anti-tumorigenic responses involving cytokine production and cytotoxic cell death (28).

It should be noted that our nomogram model does not include the plasma EBV DNA concentration. Although this factor has been considered as a potential prognosticator of NPC (29), its significance in terms of metastatic NPC remains uncertain. Additionally, many medical centers do not routinely detect the EBV DNA concentration, for which a globally standardized methodology has not been determined (6). Regarding our study, our hospital began to measure plasma EBV DNA concentrations in 2014; accordingly, this information was not available for roughly half of our cohort. Nonetheless, we admit that the exclusion of plasma EBV DNA is a limitation of our nomogram.

To the best of our knowledge, ours is the first nomogram constructed to estimate the survival of patients with synchronous and metachronous metastases of NPC. This easily used scoring system will allow clinicians and patients to perform individualized survival predictions, and the identification of subgroups of patients with different survival risks may have an impact on the selection of therapeutic regimens or care. Furthermore, our nomogram may help clinicians to address the controversial issue of screening for patients requiring additional or more intensive follow-up and could provide information useful for patient stratification in the context of a clinical investigation. Finally, our nomogram represents a more precise prognostic model when compared with the TNM staging system and some previous prognostic models.

Despite these strengths, our study has some limitations of note. First, this was a retrospective study involving a limited number of patients at a single center. A continue study with a larger patient population and external verified cohort is currently carried out by our team. Second, we used the previous version of the AJCC staging system (2009) in this study. However, in the latest version of AJCC (2017), there has only been moderate changes in the T staging and basically no relevant changes to the N and M staging. Additionally, although the internal calibration indicated a good predictive ability, the C-index for OS prediction was 0.824 (95% CI, 0.74–0.91); in other words, an external cohort is required to validate the usefulness of this model. We encourage additional prospective data collection, broader geographic recruitment and the incorporation of some other factors to improve this model.

In conclusion, we have established a novel nomogram predictive of survival in patients with distant metastases of NPC. Notably, each factor included in our nomogram is easily obtained at most hospitals. Using this model, physicians could precisely estimate the survival of individual patients and identify subgroups of patients requiring specific therapeutic strategies. Prospective randomized studies to validate this nomogram are warranted.

## Ethics Statement

The institutional review board of the First Affiliated Hospital of Xiamen University had approved this study.

## Author Contributions

LZ: Study concept and design, acquisition and analysis of patient data, drafting of the manuscript; QiuL: Acquisition and analysis of patient data, drafting of the manuscript; JG: Acquisition and analysis of patient data, software; HZ: Formal analysis, software; HC: Conceptualization, writing—review and editing; QinL: Study concept and design, funding acquisition, writing—review.

### Conflict of Interest Statement

The authors declare that the research was conducted in the absence of any commercial or financial relationships that could be construed as a potential conflict of interest.
